# Revealing the Potential of a Chimaera: a Peptide‐Peptide Nucleic Acid Molecule Designed To Interact with the SARS‐CoV‐2 Nucleocapsid Protein

**DOI:** 10.1002/anie.202420134

**Published:** 2025-02-14

**Authors:** Angela Sofia Tino, Michael Quagliata, Marco Schiavina, Lorenzo Pacini, Anna Maria Papini, Isabella C. Felli, Roberta Pierattelli

**Affiliations:** ^1^ Department of Chemistry “Ugo Schiff” University of Florence Via della Lastruccia 3–13 50019 Sesto Fiorentino Florence Italy; ^2^ Magnetic Resonance Center (CERM) University of Florence Via Luigi Sacconi 6 50019 Sesto Fiorentino Florence Italy; ^3^ Interdepartmental Research Unit of Peptide and Protein Chemistry and Biology (PeptLab) University of Florence Via della Lastruccia 13 50019 Sesto Fiorentino Florence Italy

**Keywords:** Peptide-Peptide Nucleic Acid, chimeric molecule, RNA-protein interaction, NMR, intrinsically disordered protein regions

## Abstract

Numerous RNA‐binding proteins have modular structures with folded domains and intrinsically disordered regions, making their atomic characterization difficult. This severely limits the investigation of their modalities of interaction as well as the evaluation of possible ways to interfere with this process. We report herein a rational strategy for the design and synthesis of a ligand able to interfere with the protein function, monitoring the interaction through solution nuclear magnetic resonance spectroscopy. Our approach employs a chimaera composed of two different fragments, a peptide and a peptide‐nucleic acid, allowing to incorporate in the resulting molecule key features to address RNA‐protein interactions. Focusing on two constructs of the Nucleocapsid protein from SARS‐CoV‐2, the globular N‐terminal domain and a more extended one comprising also two flanking intrinsically disordered regions, we demonstrate the enhanced affinity of the designed peptide‐peptide nucleic acid chimaera for the protein compared to a related peptide lacking π–π stacking contributions within the chain. Furthermore, we emphasize the increasingly recognized relevant and synergistic role of the intrinsically disordered regions in protein‐ligand interaction.

## Graphical abstract

Can a chimeric molecule mimic RNA and bind an RNA‐binding protein? We designed and synthesized a ligand with a dual nature within the same sequence: a pure peptide component and a peptide nucleic acid one. This chimaera is able to target the construct of the SARS‐CoV‐2 Nucleocapsid protein, which comprises the RNA‐binding globular domain and the two flanking disordered regions.

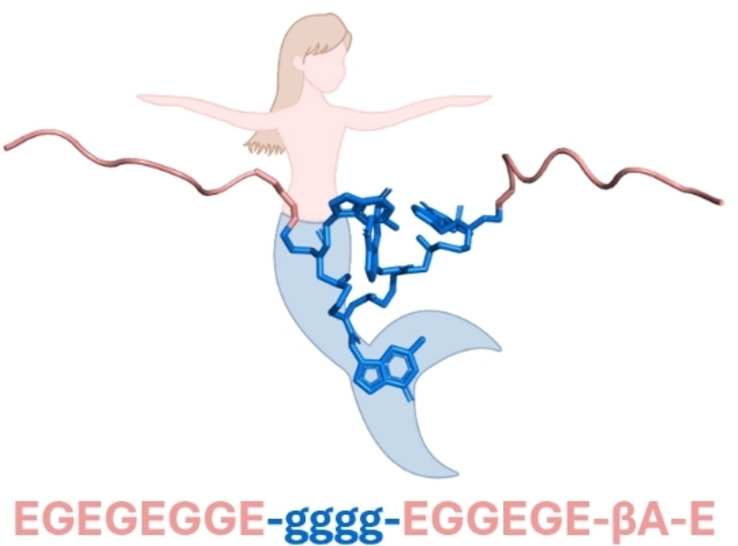

SARS‐CoV‐2 has attracted biomedical research, and its proteins have been largely scrutinized to find ways to interfere with its replication machinery. Among them, the Spike protein is being studied for both therapeutic and diagnostic purposes and is certainly the most characterized protein of this virus.[^1^, ^2^, ^3^, ^4^, ^5^, ^6^, ^7^] Nevertheless, other viral proteins play a key role in replication, such as the Nucleocapsid protein, also called N protein, which is responsible for binding viral RNA. An open question deals with the structural features of the N protein, a highly conserved protein, which is too complex to be fully structurally characterized.^[8]^ Although challenging, the possibility to acquire deeper insights into viral RNA interactions and to disrupt its primary function could be crucial for preventing infections. The complexity of studying and characterizing this protein structure stems from its modular organization, which includes two globular domains and three large intrinsically disordered regions (IDRs), preventing its crystallization. The most extensively studied domain of this protein is the N‐terminal globular domain NTD (44–180) that is hypothesized to host the main site of interaction with the viral RNA.[^9^, ^10^, ^11^] The C‐terminal domain CTD (247–364) also plays a crucial role in protein function, as it is involved in the dimerization of the full construct as well as in protein‐RNA interactions.[^12^, ^13^] However, a definitive model depicting the interaction between the N protein and the viral genome still remains elusive.^[14]^ Previous modelling studies have identified a plausible RNA‐binding site in the region of the NTD (44–180) connecting a flexible and positively charged protein segment, often referred to as the “basic finger”, with a globular core rich in hydrophobic and aromatic residues, commonly known as “aromatic palm”. This putative site aligns well with the characteristics of the RNA chain and the non‐covalent interactions driving its binding with the protein.^[15]^

We herein present a rational approach to design and synthesize a molecule specifically tailored to target this RNA‐binding protein and to monitor the interaction exploiting solution nuclear magnetic resonance (NMR) spectroscopy. To mimic RNA, we engineered a chimaera, such as a Peptide‐Peptide Nucleic Acid (P‐PNA) molecule that allowed to incorporate essential binding features, including π–π stacking from the PNA building blocks^[16]^ and additional crucial contributions in the peptide component.

In particular, the sequence of the designed chimaera is: Ac‐Glu‐Gly‐Glu‐Gly‐Glu‐Gly‐Gly‐Glu‐gggg‐Glu‐Gly‐Gly‐Glu‐Gly‐Glu‐(β‐Ala)‐Glu (EGEGEGGEggggEGGEGE(β‐Ala)E) (Figure 1).

**Figure 1:**
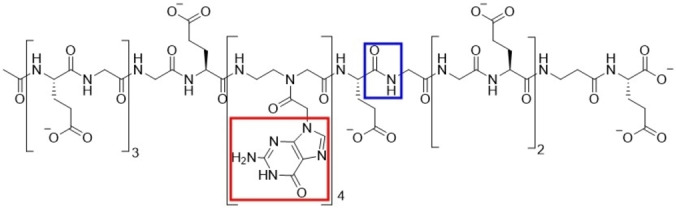
The chimaera that serves as both a nucleic acid and a peptide molecule. As highlighted into the colored boxes, the P‐PNA molecule mimics the nucleic acid through the insertion of purine bases (red box) on the side chain along a peptide‐like backbone (blue box). The latter allows for direct inclusion of amino acid functional groups.

The peptide part of the chimaera, characterized by repetitive units of Gly and Glu residues, can interact with the basic and highly flexible segments of the protein, and is useful to increase the solubility of the molecule in water solutions. At the same time, the PNA building blocks involving guanine bases (g) can contribute to form π–π stacking interactions with the structured and aromatic palm.[^17^, ^18^] The chimaera was synthesized in solid‐phase using an Fmoc/t‐Bu orthogonal protection strategy following the protocol detailed in the Supporting Information. The first 8 amino acids were coupled using an induction heating assisted automated synthesizer^[19]^ taking advantage also of a capping step after peptide bond formation. The deprotection step was performed using a solution of 20 % piperidine in DMF at 90 °C for 1 min while the coupling step was performed using a solution of Oxyma pure/DIC in DMF for 2 min at 90 °C. The remaining residues were coupled manually on resin at room temperature. In particular, the deprotection step was performed using the same piperidine solution for 5 + 10 min while the coupling step was carried out using PyOxym as activator and DIPEA as a base for 30 min. After each coupling step, the N‐terminal function, which did not react was capped with a solution of 10 % Ac_2_O in DMF to simplify the final purification step (see the Supporting Information for more details). The synthetic chimaera was thus tested against two constructs of the N protein: the N‐terminal globular domain NTD (44–180) and a longer construct containing also the two IDRs adjacent to the NTD (44–180), namely NTR (1–248). The peptide component at both termini of the chimaera was specifically designed to maintain the chain flexibility, enabling interaction with the IDRs and facilitating the assessment of their synergistic role during the binding process. Indeed, these have been shown to be important for interaction with genomic RNA fragments.[^9^, ^14^, ^20^, ^21^, ^22^, ^23^, ^24^] In particular, the flexible region IDR2 (181–248) showed two major involved regions: the region (177–203) rich in serine and arginine residues (SR‐rich), characterized by a high positive charge, and a region (216–225) rich in leucine residues (poly‐L) presenting a helical propensity within the IDR itself.[^22^, ^24^]

The interaction was monitored by conducting solution NMR titrations and observing the changes in cross peaks position and/or intensity in 2D ^1^H‐^15^N NMR spectra, which allowed to identify the most perturbed regions in the two protein constructs. As we added up to two equiv. of the P‐PNA to the NTD (44–180) construct (Figure 2), we observed spectral changes that indicated an interaction taking place in a fast exchange regime on the NMR time scale. Analysis of the HN chemical shifts revealed that the residues most affected by the interaction, in terms of chemical shift perturbations, predominantly cluster within three main regions, as reported in Figure 2B and 2C, very similar to those identified when interacting with RNA.^[15]^

**Figure 2:**
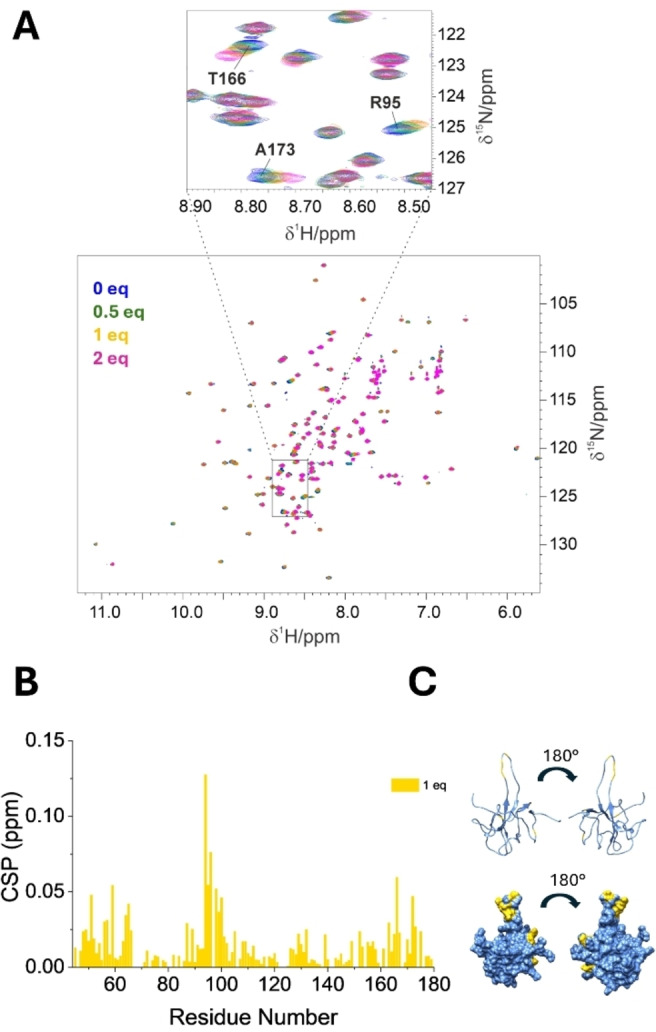
NMR titration of NTD (44–180) with P‐PNA. Panel A displays the superimposition of the reference ^1^H‐^15^N HSQC spectrum (in blue) of NTD (44–180) with spectra recorded after addition of 0.5, 1, and 2 equiv. of P‐PNA (shown in green, yellow, and magenta, respectively), along with a zoomed‐in view of a particularly perturbed region. In panel B, the combined ^1^H and ^15^N (CSP) occurring upon the addition of 1 equiv. of the P‐PNA are reported, highlighting three main affected regions of the protein construct. In panel C, the most perturbed regions are visualized in yellow on the protein frame (PDB 6YI3).

The role of the two disordered regions flanking the NTD (44–180) construct was studied by investigating the interaction of the P‐PNA chimaera with the NTR (1–248) construct under the same experimental conditions. The presence of the two disordered regions and the globular domain within the same construct allowed to investigate simultaneously their role in the interaction but rendered the NMR spectra more difficult to analyze (Figure 3), featuring a combination of signals with different properties (narrow, intense, and crowded for the intrinsically disordered regions; broad, weak and dispersed for the globular domain). Therefore, to access atom‐resolved information on both domain types (globular and intrinsically disordered domains) multiple experiments were conducted, including FAST ^1^H‐^15^N HSQC,^[25]^ SOFAST ^1^H‐^15^N HMQC,^[26]^ and ^1^H‐^15^N BEST‐TROSY,^[27]^ as described in the Supporting Information, to optimize acquisition parameters for the two different types of NMR signals.

**Figure 3:**
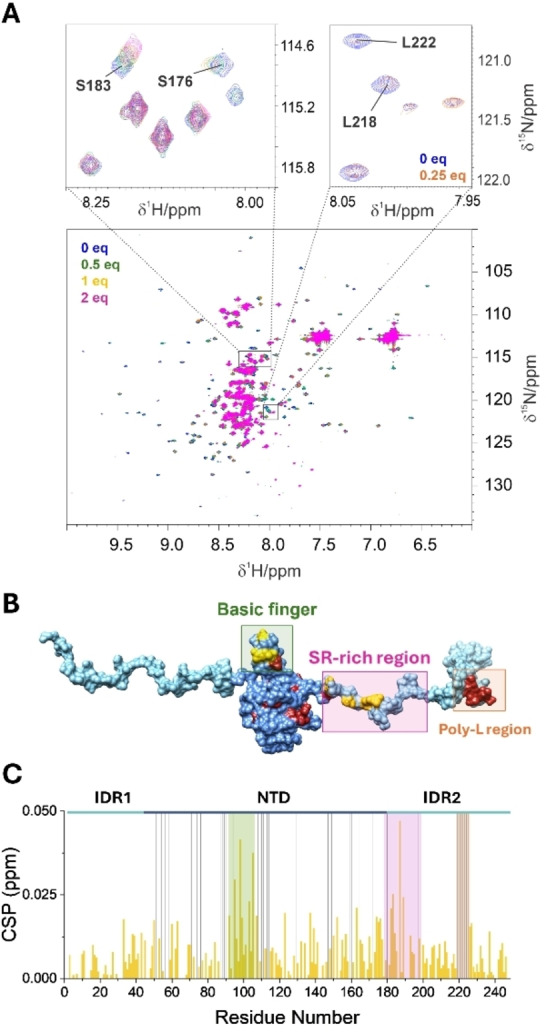
NMR titration of NTR (1–248) with P‐PNA. Panel A shows the superimposition of the reference spectrum of NTR (1–248) (in blue) with the spectra recorded after addition of 0.5, 1, and 2 equiv. of P‐PNA (in green, yellow, and magenta, respectively). The two zoomed‐in‐view show two particularly involved regions of IDR2 (181–248): the SR‐rich region, which shows the highest CSP values (on the left), and the poly‐L region (on the right) that shows several cross peaks disappearing at only 0.25 equiv. of ligand added (orange). The two regions are highlighted also on the protein frame in panel B showing the most perturbed residues mainly located on the basic finger of NTD (44–180), SR‐rich region, and poly‐L region of IDR2 (181–248). In panel C, the histograms report the ^1^H‐^15^N CSP against the residue number upon the addition of 1 equiv. of P‐PNA. Additionally, the gray lines highlight the peaks that disappear, and the colored boxes in panel B underline the basic finger (green), the SR‐rich region (magenta) and the poly‐L region (orange).

As reported in Figure 3, the residues belonging to the IDRs, and those of IDR2 (181–248) in particular, are among the first interactors with the P‐PNA. The most perturbed residues are found in the SR‐rich region (177–203, pink rectangle in Figure 3C), suggesting an interaction driven by electrostatic attractions between the positively charged arginine side chains and the negatively charged residues of the chimaera. In addition, several peaks corresponding to the residues of the poly‐L region (216–225, orange rectangle in Figure 3C) disappeared immediately after the addition of only 0.25 equiv. of the chimaera. These observations indicate that the regions that are most affected by the interaction are similar to those identified when studying the interaction of NTR (1–248) with RNA fragments.[^22^, ^24^] In the presence of IDRs, the major chemical shift perturbations (CSPs) on the NTD (44–180) region were identified once again in the basic finger of NTD (44–180) (light green rectangle in Figure 3C). However, for what concerns the globular domain in the NTR (1–248) construct, CSP is not the main indicator of the interaction. Indeed, several peaks of NTD (44–180) decrease in intensity or disappear completely (grey bars in Figure 3C) upon the addition of the chimaera molecule. It is worth noting that most of these residues are located in regions previously identified as interaction sites in the isolated NTD (44–180). Moreover, the CSP of residues belonging to the IDRs clearly demonstrate a fundamental contribution of disordered regions in the interaction. In particular, we hypothesize both direct and indirect effects on protein regions, possibly involving internal rearrangements of pre‐existing intramolecular interactions, as already discussed in the literature.^[23]^ This could explain the observed differences in peaks behavior among residues from different domains of the protein construct.

The concept of using the P‐PNA chimaera as interactor represents an evolutionary step that originates from our interest in protein‐polyanionic peptide interactions. Our primary idea was to have a “modular” structure capable of adapting to the heterogeneous structural and dynamic nature of the N protein.

This design firstly resulted in the peptide sequence EGEGEGGLLELALELLGGEGE(β‐Ala)E, characterized by a predicted secondary structural propensity promoted by a high concentration of leucine residues in the middle (LLELALELL) and two more flexible ends promoted by repetitions of GE residues retained in the chimeric molecule (EGEGEGG and GGEGE(β‐Ala)E). This initial study was conducted as a proof‐of‐concept and was subsequently extended to investigate the chimeric molecule (Figure 4).

**Figure 4:**
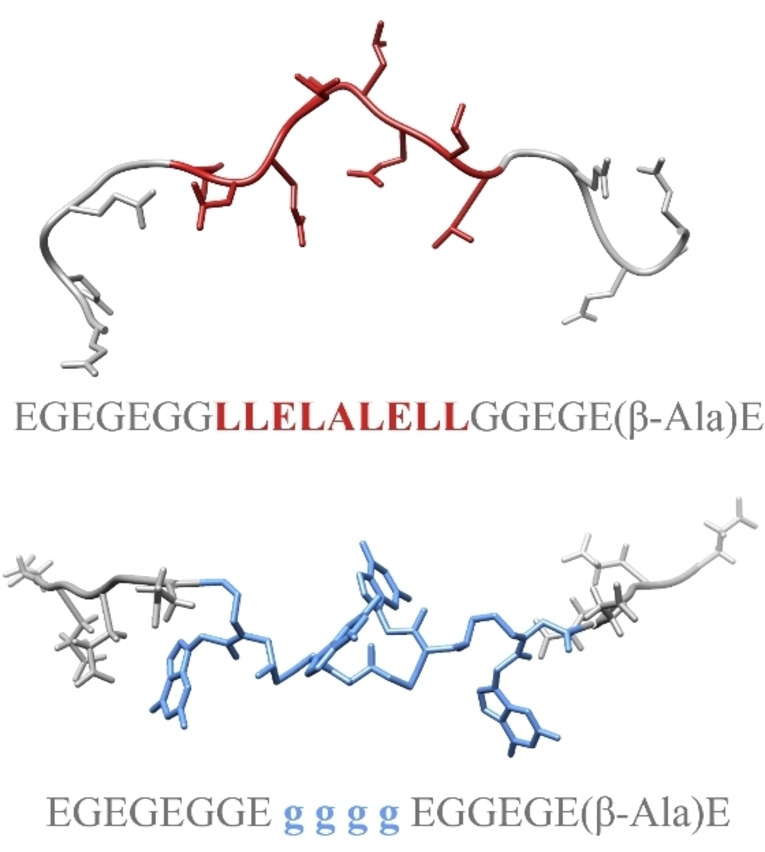
The rationale to develop the P‐PNA molecule. The two termini of the peptide chain (on the top) were kept preserving the electrostatic contribution given by glutamic acid residues and the high flexibility provided by glycine residues. The central part of the peptide (red) was replaced with four PNA building blocks (blue), with guanine as purine base to introduce a strong aromatic component mimicking the nature of the viral RNA (bottom).

The synthetic peptide EGEGEGGLLELALELLGGEGE(β‐Ala)E (peptide P) used in NMR titration in solution with the NTD (44–180) construct of the protein, enabled to assess its interaction capability. We monitored the interaction using 2D ^1^H‐^15^N HSQC spectra, which revealed a clear pattern of fast exchange on the NMR timescale and enabled the identification of the most affected amino acids within the protein construct. Comparative analysis clearly highlights the P‐PNA chimeric molecule superior affinity over the pure peptide precursor affirming our innovative strategy to use PNA building blocks (Figure 5). We estimated the binding affinities (in terms of dissociation constant, K_d_) for the most perturbed residues of NTD (44–180) in both titrations with the peptide and with the chimaera. Figure 5 reports the fitting obtained for the cross peak of residue 94, which is located in a protein region known to be crucial for gRNA binding. The average of the dissociation constants obtained for the interaction between NTD (44–180) and P‐PNA, is in the range of hundred micromolar, demonstrating enhanced affinity with respect to the peptide. This value, despite larger, is not significantly distant from those reported in the literature for studies conducted with gRNA fragments.[^9^, ^11^, ^24^, ^29^] The K_d_ values were calculated using the observed CSPs of the corresponding cross peaks upon addition of the ligand according to the equation reported in the Supporting Information.

**Figure 5:**
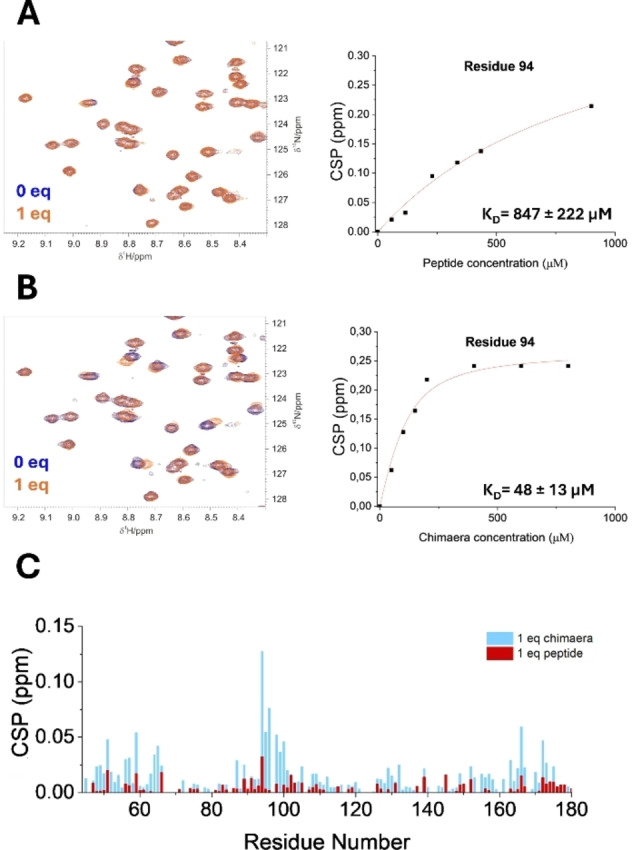
Comparison of binding capability of the P‐PNA chimaera and the peptide P with NTD (44–180) monitored by NMR. In panel A, the NMR spectra of the titration between NTD (44–180) and the peptide are shown, while panel B reports the analogous data for the titration of the NTD (44–180) with the chimaera. Both spectra depict the superimposition of a reference spectrum in blue with the one corresponding to the addition of 1 equiv. of ligand, in orange. The image clearly shows that CSP for the same residues are more pronounced in the case of the chimaera adduct; as an example, the K_d_ calculated for residue 94 underscores the higher affinity of the chimaera for the protein construct. Panel C reports the superimposition of the CSP values versus the residue number for the two cases (in light blue 1 equiv. of P‐PNA chimaera added and in red 1 equiv. of the peptide added).

The PNA incorporation, which includes the aromatic contribution from the nucleobases, steered our investigation towards promising outcomes. Indeed, as evidenced by the comparison of the plots in Figure 5, the most perturbed residues of the protein are predominantly localized in the same regions. However, the chemical shift perturbation values are clearly higher for the chimeric molecule as a ligand.

In conclusion, we outlined a strategy for a rational approach to design a molecule that incorporates all the characteristics necessary for interaction with RNA binding proteins and ideally interfere with protein‐nucleic acid interactions. Specifically, we demonstrated that the P‐PNA chimaera exhibits higher affinity for an RNA‐binding protein, such as the nucleocapsid protein of SARS‐CoV‐2, compared to a pure designed peptide. The power of this study lies in the design of a chimeric molecule that is synthetically affordable. This PNA‐based molecule represents a unique example of an N protein binder, diverging from the nucleic acid binders commonly found in the literature.^[16]^ In targeting the protein, the inclusion of a peptide component is essential to harness electrostatic contributions absent in a standalone PNA molecule, which permits to mimic the RNA nature and the driving forces of its interaction with an RNA‐binding protein. While the binding affinity of P‐PNA is lower than that of some oligonucleotides of similar length,[^9^, ^15^] this work serves as a proof‐of‐concept, demonstrating that rationally designed synthetic molecules can successfully target the putative RNA‐binding site of the N protein. From a structural biology standpoint, we have reaffirmed the key protein regions implicated in interactions involving the NTD (44–180) protein construct. Furthermore, we have confirmed the increasingly recognized synergistic importance of the IDRs in these interactions, particularly emphasizing the well‐known domains such as the SR‐rich and L‐rich regions within the IDR2 (181–248). By extending the protein construct involving also the folded domain essential for the viral assembly and RNA binding, i.e. CTD (247–364), in future studies it will be possible to understand how synergistic interactions between domains can modulate binding specificity and stability.[^12^, ^13^] In this context, we developed an original strategy to design and synthesize putative ligands, which enables the assessment of key features of protein interactions from a novel perspective. This approach helps to identify the essential aspects driving these processes, including those that modulate the onset of liquid‐liquid phase separation, a key feature in the context of RNA‐binding proteins.[^20^, ^24^, ^30^, ^31^, ^32^] Finally, given this approach based on a rational design, improvements in molecule affinity for the protein and in vivo bioavailability could in a near future significantly impact drug discovery and the study of related proteins binding nucleic acids.

## Conflict of Interests

The authors declare no conflict of interest.

## Supporting information

As a service to our authors and readers, this journal provides supporting information supplied by the authors. Such materials are peer reviewed and may be re‐organized for online delivery, but are not copy‐edited or typeset. Technical support issues arising from supporting information (other than missing files) should be addressed to the authors.

Supporting Information

## Data Availability

The data that support the findings of this study are available from the corresponding authors upon reasonable request.
