# Supplementary material for: Revealing the Potential of a Chimaera: a Peptide‐Peptide Nucleic Acid Molecule Designed To Interact with the SARS‐CoV‐2 Nucleocapsid Protein
Source: Angew Chem Int Ed Engl. 2025 Feb 14;64(11):e202420134. doi: 10.1002/anie.202420134 (PMC11891622; doi:10.1002/anie.202420134)
Supplement: Supplementary file 1 — Supporting Information [file ANIE-64-e202420134-s001.pdf]

Angewandte Chemie
Eine Zeitschrift der Gesellschaft Deutscher Chemiker

Supporting Information

Revealing the Potential of a Chimaera: a Peptide-Peptide Nucleic Acid Molecule Designed To Interact with the SARS-CoV-2 Nucleocapsid Protein

A. S. Tino, M. Quagliata, M. Schiavina, L. Pacini, A. M. Papini, I. C. Felli*, R. Pierattelli**

Supporting Information

Revealing the Potential of a Chimaera: a Peptide-Peptide Nucleic Acid Molecule Designed To Interact with the SARS-CoV-2 Nucleocapsid Protein

Angela Sofia Tino[a,b] Michael Quagliata[a,c], Marco Schiavina[a,b], Lorenzo Pacini[a,c], Anna Maria Papini*[a,c], Isabella C. Felli*[a,b] and Roberta Pierattelli*[a,b]

[a] Ms. A.S. Tino, Mr. M. Quagliata, Dr. M. Schiavina, Mr. L. Pacini, Prof. Dr. A.M. Papini, Prof. Dr. I.C. Felli, Prof. Dr. R. Pierattelli
Department of Chemistry "Ugo Schiff", University of Florence, Via della Lastruccia 3-13, 50019 Sesto Fiorentino, Florence, Italy
E-mail: annamaria.papini@unifi.it; felli@cerm.unifi.it, roberta.pierattelli@unifi.it

[b] Ms. A.S. Tino, Dr. M. Schiavina, Prof. Dr. I.C. Felli, Prof. Dr. R. Pierattelli
Magnetic Resonance Center (CERM), University of Florence, Via Luigi Sacconi 6, 50019 Sesto Fiorentino, Florence, Italy

[c] Mr. M. Quagliata, Mr. L. Pacini, Prof. Dr. A.M. Papini
Interdepartmental Research Unit of Peptide and Protein Chemistry and Biology (PeptLab), University of Florence, Via della Lastruccia 13, 50019 Sesto Fiorentino, Florence, Italy

EXPERIMENTAL SECTION

Reagents. All Fmoc-protected amino acids, *N*,*N′*-diisopropylcarbodiimide (DIC), OxymaPure® (ethyl cyanohydroxyiminoacetate) were purchased from Iris Biotech GmbH (Marktredwitz, Germany). The PNA monomer Fmoc-G(Bhoc)-OH was purchased from ASM Research Chemicals (Hannover, Germany). PyOxym was a gift from Luxembourg Biotech (Rehovot, Israel). Tentagel® S RAM resin was purchased from Rapp Polymere (Tuebingen, Germany). Peptide-synthesis grade *N,N*-dimethylformamide (DMF) and acetonitrile (ACN) were purchased from Carlo Erba (Milano, Italy). Dichloromethane (DCM), trifluoroacetic acid (TFA), triisopropylsilane (TIS), acetic anhydride (Ac_2O), and piperidine were purchased from Sigma-Aldrich (Milano, Italy).

Synthesis of the peptide Ac-EGEGEGGLLELALELLGGEGE-βA-E. The peptide was synthesized by Induction-assisted Solid-Phase Peptide Synthesis (I-SPPS) following the Fmoc/*t*Bu orthogonal protection strategy, using the PurePep™Chorus™ automated peptide synthesizer (Gyros Protein Technologies, Uppsala, Sweden). Tentagel® S RAM resin was used (loading 0.23 mmol/g). The following Fmoc-amino acids were used: Fmoc-βAla-OH, Fmoc-L-Glu(OtBu)-OH, Fmoc-Gly-OH, Fmoc-L-Leu-OH, Fmoc-L-Ala-OH. Fmoc deprotections were performed with a solution of 20% piperidine in DMF for 60 seconds at 363 K. Peptide assembly was performed by repeating the SPPS standard coupling cycle for each amino acid, using Fmoc-protected amino acids (5 equiv), OxymaPure® (5 equiv), and DIC (5 equiv) dissolved in DMF for 120 seconds at 363 K. The washing steps were performed using a mixture of AcOEt:DMSO 8:2 (v:v). All the Fmoc-L-Glu(OtBu)-OH and Fmoc-βAla-OH were coupled twice. The *N*-acetylation was performed using a solution of 10% Ac_2O in DMF for 10 minutes at room temperature. Final cleavage and side-chain deprotections were performed using a mixture of TFA/TIS/H_2O/EDT (94:3:1.5:1.5, v:v:v:v) at room temperature. After 2 hours the resin was filtered off. The peptide was precipitated with cold Et_2O, centrifuged, and lyophilized. The crude peptide was purified by Reverse-Phase Flash Liquid Chromatography on an Isolera One Flash Chromatography (Biotage, Uppsala, Sweden) using a SNAP Ultra C18 column (12 g) at 12 mL/min as solvent systems H_2O (MilliQ) and ACN. Analytical characterization of the peptide was performed by HPLC using a Waters ACQUITY HPLC coupled to a single quadrupole ESI-MS (Waters® ZQ Detector, Waters Milford, MA, USA) supplied with a BEH C18 (1.7 µm, 2.1 × 50 mm) column at 308 K, at 0.6 mL/min using solvent systems A (0.1% TFA in H_2O) and B (0.1% TFA in ACN). Gradient elution was performed with a flow of 0.6 mL/min. The analytical data, the chromatograms, and mass spectrometry spectra are reported in Figure S1.

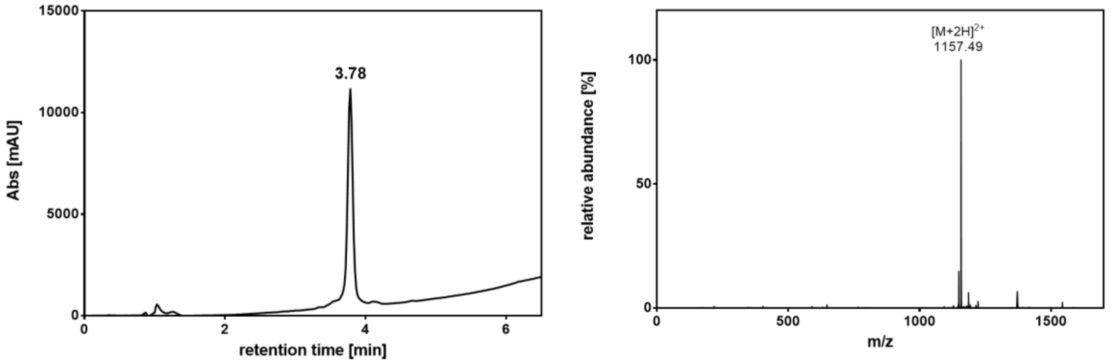

Figure S1. RP-UHPLC traces of the peptide Ac-EGEGEGGLLELALELLGGEGE-βA-E. Conditions: C18 column Waters Acquity CSH (130 Å, 1.7 µm, 2.1 × 50 mm); temperature, 318 K; flow, 0.5 mL/min; eluents, 0.1% (v/v) TFA in H_2O (A) and 0.1% (v/v) TFA in ACN (B); λ, 215 nm; gradient, 30−90% B in 5 min. Rt = 3.78 min, Ac-EGEGEGGLLELALELLGGEGE-βA-E (left panel). ESI-MS spectrum Ac-EGEGEGGLLELALELLGGEGE-βA-E. ESI-MS (m/z): $[M+2H]^{2+}$ 1157.49 (found), 1157.25 (calcd) (right panel).

Synthesis of the chimaera Ac-EGEGEGGE-gggg-EGGEGE-βA-E . The chimaera was synthesized by Induction-assisted Solid-Phase synthesis as reported above until the insertion of the first Fmoc-protected PNA monomer in position 12. Then the synthesis proceeded at room temperature. Fmoc deprotections were performed with a solution of 20% piperidine in DMF for 10 + 5 minutes. The coupling steps were performed using Fmoc-protected amino acids or Fmoc-G(Bhoc)-OH (5 equiv), PyOxim (5 equiv), and DIPEA (5 equiv) dissolved in DMF for 30 minutes. The washing steps were performed using a mixture of AcOEt:DMSO 8:2 (v:v). The N-acetylation was performed using a solution of 10% Ac_2O in DMF for 10 minutes at room temperature. Final cleavage and side-chain deprotections were performed using a mixture of TFA/TIS/H_2O/EDT (94:3:1.5:1.5, v:v:v:v) at room temperature. After 2 hours the resin was filtered off. The chimaera was precipitated with cold Et_2O, centrifuged, and lyophilized. The crude chimaera was purified by Reverse-Phase Flash Liquid Chromatography on an Isolera One Flash Chromatography (Biotage, Uppsala, Sweden) using a SNAP Ultra C18 column (12 g) at 12 mL/min as solvent systems H_2O (MilliQ) and ACN both with 0.1% TFA (v/v). Analytical characterization of the compound was performed by HPLC using a Waters ACQUITY HPLC coupled to a single quadrupole ESI-MS (Waters® ZQ Detector, Waters Milford, MA, USA) supplied with a BEH C18 (1.7 µm, 2.1 × 50 mm) column at 308 K, at 0.6 mL/min using solvent systems A (0.1% TFA in H_2O) and B (0.1% TFA in ACN).

Gradient elution was performed with a flow of 0.6 mL/min. The analytical data, the chromatograms, and mass spectrometry spectra are reported in Figure S2.

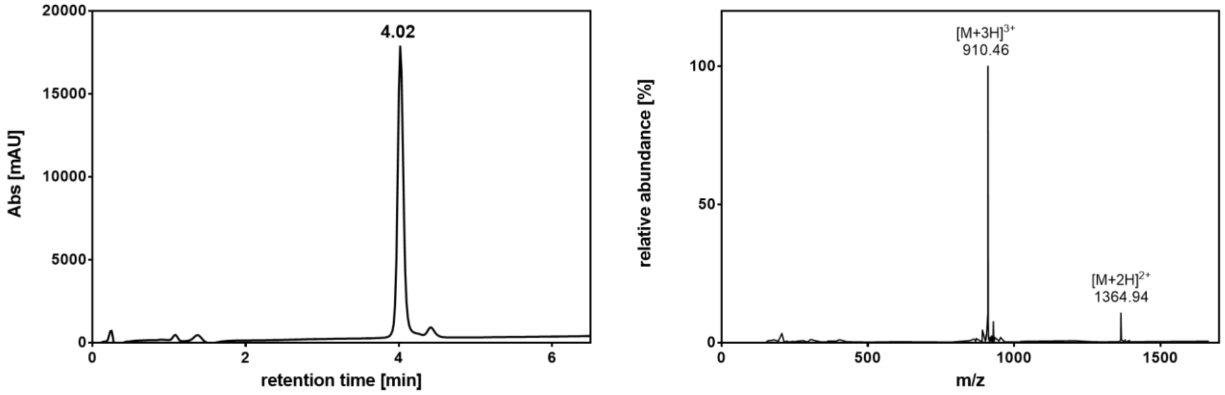

Figure S2. RP-UHPLC traces of the peptide Ac-EGEGEGGE-gggg-EGGEGE-βA-E (left panel). Conditions: C18 column Waters Acquity CSH (130 Å, 1.7 µm, 2.1 × 50 mm); temperature, 318 K; flow, 0.5 mL/min; eluents, 0.1% (v/v) TFA in H_2O (A) and 0.1% (v/v) TFA in ACN (B); λ, 215 nm; gradient, 3−30% B in 5 min. Rt = 4.02 min, Ac-EGEGEGGE-gggg-EGGEGE-βA-E. ESI-MS spectrum Ac-EGEGEGGE-gggg-EGGEGE-βA-E. ESI-MS (m/z): $[M+3H]^{3+}$ 910.46 (found), 910.17 (calcd) (right panel).

Analytical characterization of the synthetic sequences. The sequences were characterized by RP-HPLC Alliance Chromatography system (Waters, Milford Massachusetts, USA) with a BEH C18 (1.7 µm, 2.1× 50 mm) column at 308 K, 0.6 mL/min, coupled to a single quadrupole ESI-MS Micromass ZQ (Waters, Milford Massachusetts, USA).

	Sequence	*HPLC gradient (%B) R_f (min)*[a]	*HPLC purity(%)*	*Yield (%)*[b]	*ESI-MS (m/z) found (calcd)*
**Peptide (P)**	Ac-EGEGEGGLLELALELLGGEGE-βA-E	30-90 3.78	96	26	1157.49 (1157.25)[c]
**Chimaera (P-PNA)**	Ac-EGEGEGGE-gggg-EGGEGE-βA-E	03-30 4.02	95	20	910.46 (910.17)[d]

Table S1: Analytical characterization of the synthetic peptide and chimaera. Eluents: 0.1% (v/v) TFA in H_2O (A) and 0.1% (v/v) TFA in ACN (B), λ 215 nm. Gradient times: 5 minutes; [b]calculated as the ratio of obtained mass to theoretical mass. ESI-MS: detected as [c]$[M+2H]^{2+}$, [d]$[M+3H]^{3+}$.

NMR Samples. The uniformly ^{15}N labelled NTD^{44-180} and the NTR^{1-248} samples were prepared as previously described [33,34] to reach a final concentration of 100 µM and 70 µM respectively in 600 µL volume.

The peptide and the chimaera NMR experiments were acquired using two samples at a concentration of 0.6 mM in 320 µL volume and 1.0 mM in 600 µL volume, respectively.

All the NMR experiments were carried out in the same NMR buffer: 12.5 mM potassium phosphate, 50 mM potassium chloride (KCl), 100 µM 2,2',2'',2'''-(ethane-1,2-diyldinitrilo)tetraacetic acid (EDTA), 0.03% sodium azide (NaN_3) at pH 6.5 in H_2O with 5% D_2O.

NMR Experiments. The spectra of the peptide and those used to follow the interaction between NTD^{44-180} and the two molecules (the peptide and the chimaera) were recorded at a spectrometer operating at 21.1 T (899.9 MHz ^1H frequency, 226.3 MHz ^{13}C frequency, 91.2 MHz ^{15}N frequency) equipped with a triple resonance cryogenically cooled (TCI) probe-head optimized for inverse detected NMR experiments.

The spectra of the chimaera were recorded at a spectrometer operating at 22.3 T (950.2 MHz ^1H frequency, 238.9 MHz ^{13}C frequency, 96.3 MHz ^{15}N frequency) equipped with a TCI probe-head.

The spectra needed to follow the interaction between NTR^{1-248} and the chimaera were recorded at a spectrometer operating at 28.2 T (1200.6 ^1H frequency, 301.9 MHz ^{13}C frequency, 121.6 MHz ^{15}N frequency) equipped with a triple resonance cryogenically cooled probe-head optimized for ^{13}C direct detection (TXO).

A 1.0 mM batch of the chimaera solution was prepared in the NMR buffer and small aliquots were added to protein solution samples to reach NTD^{44-180} :chimaera ratios of 1:0.5, 1:1, 1:1.5, 1:2, 1:4, 1:6, and 1:8 and NTR^{1-248} :chimaera ratios of 1:0.25, 1:0.50, 1:0.75, 1:1, 1:1.5, 1:2, 1:4, and 1:8.

A 2.0 mM batch of the peptide solution was prepared in the NMR buffer and added to the protein solution sample as described in the "Titration strategy" session (*vide infra*) to reach NTD^{44-180}:peptide ratios of 1:0.6, 1:1.2, 1:2.3, 1:3.3, 1:4.3, 1:9, and 1:20.

The interaction between the NTD^{44-180} construct and the two molecules was followed at 298 K, exploiting a series of 2D sensitivity improvement HN HSQC [25] experiments. The carrier frequency for ^1H was set at 4.7 ppm; for ^{15}N, the carrier was set at 117 ppm.

The interaction between the NTR$^{1-248}$ construct and the two molecules was followed at 298 K, exploiting a series of 2D FAST HN HSQC [25], 2D SOFAST HMQC [26] and 2D BEST TROSY HN [27,36] experiments. The carrier frequency for ^{1}H was set at 4.7 ppm; for ^{15}N, the carrier was set at 118 ppm.

Standard radiofrequency pulses were used. The decoupling of ^{15}N was achieved with garp4 decoupling sequence [37] with 1 kHz radiofrequency. All gradients employed had a smoothed square shape.

To obtain the fingerprint of the unlabeled peptide and the chimaera samples, 1D excitation sculpting ^{1}H [38], 2D HSQC sensitivity improvement HN [35] and 2D sensitivity improvement HSQC HC [25] NMR experiments were recorded.

The acquisition NMR parameters are reported in Table S2.

**Sample**	**Experiment**	**Number of scans**	**Interscan delay (s)**	**Data points**		**Spectra width (Hz)**	
				F1	F2	F1	F2
Chimaera (P-PNA)	HSQC HN	64	1.08	448	4096	2315 ^{15}N (24 ppm)	23809 ^{1}H (25 ppm)
	HSQC HC	32	2.09	831	2048	14493 ^{13}C (60 ppm)	11398 ^{1}H (12 ppm)
Peptide (P)	HSQC HN	64	1.08	400	2048	3650 ^{15}N (40.0 ppm)	13158 ^{1}H (14.6 ppm)
	HSQC HC	32	1.53	384	1024	18182 ^{13}C (80 ppm)	14706 ^{1}H (16 ppm)
NTD$^{44-180}$ + Peptide	HSQC HN	16	1.08	320	2048	3649 ^{15}N (40 ppm)	13157 ^{1}H (14 ppm)
NTD$^{44-180}$ + Chimaera	HSQC HN	16	1.08	320	2048	3649 ^{15}N (40 ppm)	13157 ^{1}H (14 ppm)
NTR$^{1-248}$ + Chimaera	FAST-HSQC HN	16	1.11	800	4096	4386 ^{15}N (36 ppm)	19231 ^{1}H (16 ppm)
	SOFAST-HSQC HN	64	0.45	360	2048	4386 ^{15}N (36 ppm)	19231 ^{1}H (16 ppm)
	BEST TROSY HN	16	0.40	1536	8192	4386 ^{15}N (36 ppm)	19231 ^{1}H (16 ppm)

Table S2: Acquisition parameters of NMR experiments.

Titration method. The interactions, which involved the chimaera and the two different protein constructs were carried out using two 5 mm NMR tubes: a reference tube (tube 1) containing the protein in the NMR buffer and another tube (tube 2) with a batch of the molecule at 1.0 mM in the same buffer. By adding increasing volumes of the tube 2 to the tube 1, the titration points corresponding to the concentration ratios described above were obtained.

Regarding the interaction between the peptide and the NTD$^{44-180}$ construct of the protein, two tubes were used, both prepared with 600 µL of 100 µM protein in the NMR buffer. In this case, a 2.0 mM solution of the peptide was solubilized in tube 2. The tube 2 represented the last point of the titration. By mixing the contents of the two tubes in different proportions, the various titration points previously described were obtained.

Chemical Shift Perturbations (CSP) analysis. The titrations and the interactions among the protein constructs and the two different ligands were followed observing the chemical shift perturbations for each residue of the target protein in both dimensions of NMR 2D HN correlation spectra (^{1}H and ^{15}N). The equation used to combine the CSP in the two dimensions of the nuclei is [39]:

$$CSP\ (ppm) = \sqrt{(CSP(H) * CSP(H)) + (0.0997 * ((CSP(N) * CSP(N)))} \quad (1)$$

where CSP is the difference in ppm between the observed chemical shift perturbation (CSP) at the different titration points and the chemical shift value of the reference spectrum for each residue. In particular, *CSP(H)* represents the CSP perturbation value in the proton dimension while *CSP(N)* the CSP perturbation one in the nitrogenous dimension. All the plots in the figures of the main text report these calculated values.

K_d Estimation. The dissociation constant (Kd) for the interaction between the NTD construct and the two different ligands was determined through NMR spectroscopy measuring the variation of chemical shift for each peak in a series of 2D ^{1}H-^{15}N HSQC spectra recorded at increasing concentrations of the ligand. The data were fitted using the following equation[39]:

$$CSP\ (ppm) = \frac{1}{1 + \left(\frac{K_d}{C_L - 0.5\left(C_P + CL + Kd - \sqrt{(P + C_L + Kd)^2 - 4CPC_L}\right)}\right)} \cdot m \quad (2)$$

where CSP is the difference in ppm between the observed chemical shift perturbation (CSP) at the different titration points and the chemical shift value of the reference spectrum, C_P is the total protein concentration (NTD), C_L is the ligand concentration (peptide or chimaera molecule) at the different titration points, m represents the maximum expected CSP value upon the complete formation of the bound state, and K_d is the dissociation constant. The CSP values of those peaks displaying a perturbation higher than the average plus two standard deviations at 2 eq of ligand added were used as inputs in the calculation to estimate the Kd. The residues used to calculate the K_d for the NTD construct were S51, H59, I94, R95, G96, D98, G99, T166, and Y172, which are the ones highlighted in yellow in Figure 2.

For the peptide, we selected the eight most affected residues at the same equivalents, which are: S51, H59, R89, T91, I94, K102, T166, and Y172.

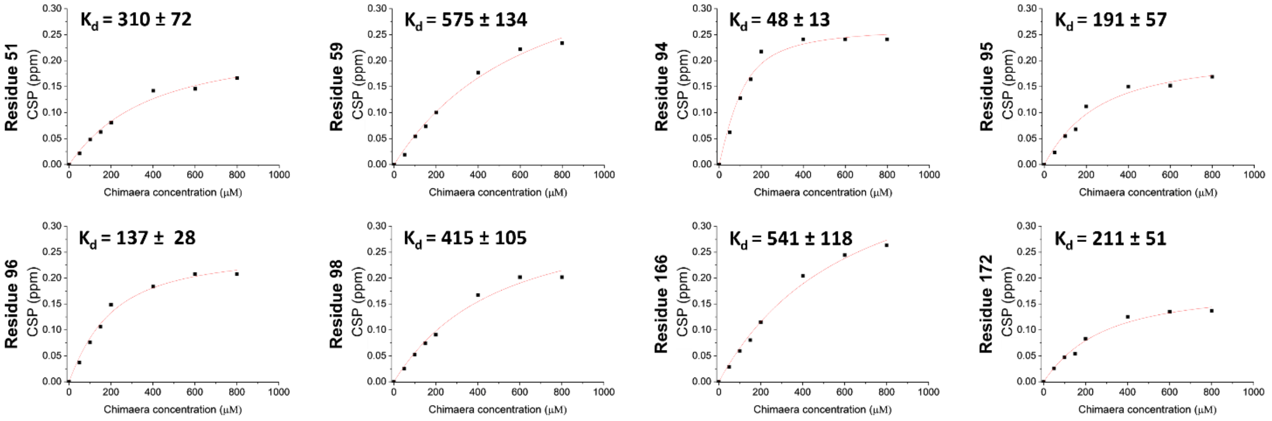

Figure S3. The measured CSP values upon addition of increasing amounts of P-PNA chimera are reported for the most affected residues (S51, H59, I94, R95, G96, D98, G99, T166, and Y172) together with the K_d estimated using equation 2. All the K_d are expressed in μM.

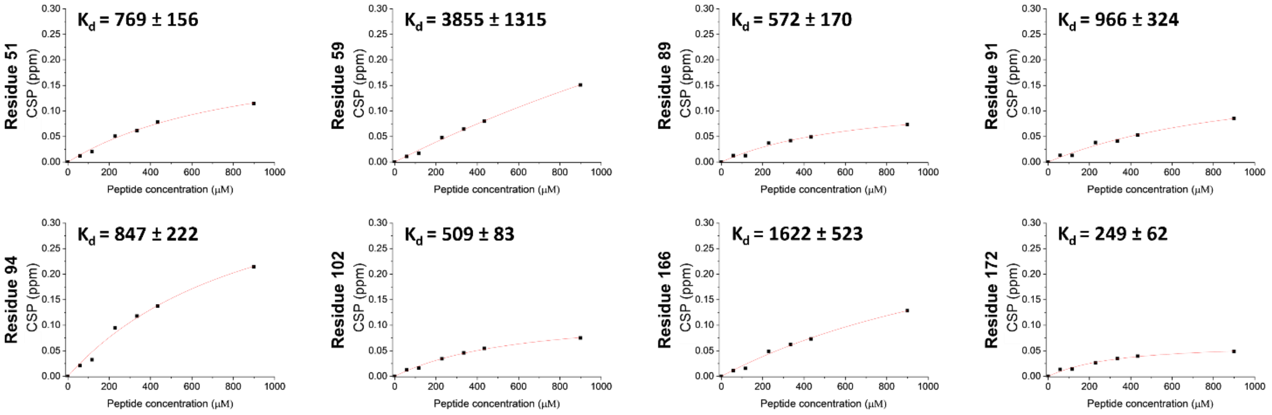

Figure S4. The measured CSP values upon addition of increasing amounts of peptide P are reported for the most affected residues (S51, H59, R89, T91, I94, K102, T166, and Y172) together with the K_d estimated using equation 2. All the K_d are expressed in μM.
